# How Temperature Influences Sleep

**DOI:** 10.3390/ijms232012191

**Published:** 2022-10-13

**Authors:** Yaqian Fan, Yuedong Wang, Pengyu Gu, Junhai Han, Yao Tian

**Affiliations:** School of Life Science and Technology, The Key Laboratory of Developmental Genes and Human Disease, Southeast University, 2 Sipailou Road, Nanjing 210096, China

**Keywords:** temperature, transient receptor potential, sleep, dorsal neuron, preoptic area

## Abstract

Sleep is a fundamental, evolutionarily conserved, plastic behavior that is regulated by circadian and homeostatic mechanisms as well as genetic factors and environmental factors, such as light, humidity, and temperature. Among environmental cues, temperature plays an important role in the regulation of sleep. This review presents an overview of thermoreception in animals and the neural circuits that link this process to sleep. Understanding the influence of temperature on sleep can provide insight into basic physiologic processes that are required for survival and guide strategies to manage sleep disorders.

## 1. Introduction

From *Drosophila* to mammals, sleep is a conservative biological behavior. While sleeping, animals are at risk of predation, but they still spend a lot of time sleeping, suggesting that sleep is important to animals. Recent studies have shown that sleep is essential for replenishing energy after activity, ensuring an optimal physical condition [1], maintaining synaptic stability [2,3,4], regulating immunity [5,6], memory consolidation [7,8], and removing neurotoxic waste [9,10]. Sleep disturbances impair the function of the sympathetic nervous system, leading to metabolic dysregulation [11]. Insufficient sleep during developmental stages can cause a smaller brain and abnormal behavior [12]. In conclusion, sleep is essential for the development, behavior, and survival of organisms. Although the molecular and neural circuits involved in sleep regulation have been extensively studied, the influence of different environmental factors on sleep is not fully understood.

In 1982, Borbely proposed a dual-system model based on previous research results on sleep and used a mathematical model to describe the sleep mechanism [13]. Borbely proposed that sleep is jointly regulated by the rhythmic system (process C) and the sleep-wake homeostatic system (process S). Circadian rhythm determines the periods of wakefulness and sleep over the 24-h daily cycle. Sleep homeostasis preserves physiologic stability; prolonged wakefulness increases the pressure to fall asleep, whereas prolonged sleep time relieves sleep pressure and promotes wakefulness [14]. Studies on the regulatory mechanisms of sleep have identified distinct neural circuits and multiple neurotransmitters that are responsible for maintaining sleep homeostasis and circadian rhythm [15,16,17,18] using model animals such as flies, zebrafish, and mice [8,19]. Based on different model animals, we are increasingly understanding the neural circuits and regulatory molecules that regulate sleep. The regulation of sleep by environmental factors remains to be explored. The brain pathways’ underlying lights impact on sleep have been examined in the current research work [20,21]. It is still unclear how temperature regulation affects sleep.

There are two commonly used methods: one is based on changes in animal sleep-related behaviors and the other is based on changes in brain waves. Five characteristics of sleep in animals include a long period of stillness, higher threshold of response to external stimuli, arousal when stimulated (to distinguish numbness or coma), a distinctive posture, and rebound following sleep deprivation [16,22]. The main criterion for identifying sleep behavior in *Drosophila* is immobility; flies that are stationary for 5 or more minutes are generally judged to be sleeping [23,24]. The simplicity of this behavior-based definition of sleep makes *Drosophila* a useful model animal for studying the the complex physiologic mechanisms involved and for identifying novel genes and neurons that regulate this process [25,26,27]. Sleep in mice is analyzed by changes in brain activity [28,29] based on brain waves, and sleep can be categorized as rapid eye movement (REM) sleep and non-REM (NREM). Based on well-established research methods and research models, the researchers explored the effect of temperature on sleep. The present work reviews the current state of knowledge regarding the neural bases of thermoreception and sleep, and the effect of temperature on sleep based on studies in *Drosophila* and mammals.

## 2. Effect of Temperature on Sleep

Changes in ambient temperature not only affect ectothermic animals, but also affect the sleep of warm-blooded animals. Many animals build nests or curl up before sleeping to ensure that their bodies stay warm. In mice and humans, sleep can be triggered when the skin warms up [30]. In hot environments, the body cools itself, promoting wakefulness and changing sleep patterns [31]. When exposed to low temperatures, the body produces heat on its own and promotes arousal [32]. *Drosophila* has a limited ability to control body temperature; therefore, variations in ambient temperature can have a strong impact on behavior. Both prolonged and sudden temperature changes can affect sleep; temperature fluctuations throughout the day are the basis of the circadian rhythm of the rest-activity cycle of *Drosophila* [33]. *Drosophila’s* sleep was shown to increase during the day and decrease at night when the ambient temperature shifted from 25 °C to 29 °C [34,35,36]; a suddenly temperature shift from 22 °C to 29 °C decreased sleep during the day and at night [34,37]. Therefore, temperature affects the sleep patterns of *Drosophila*. By monitoring the sleep of *Drosophila* species at different latitudes, it was observed that sleep time was related to annual average temperature [38]. Compared with species at low-latitudes, those living at high latitudes had a shorter sleep duration [39]. Additionally, low temperature-dependent splicing increased the transcription level of the *daywake* (*dyw*) genes, providing evidence that sleep is regulated by ambient temperature at the molecular level [40,41]. 

Unlike *Drosophila*, mammals produce heat by regulating their own metabolism and maintaining their body temperature within a restricted range. Body temperature is regulated by circadian rhythm, and core temperature begins to decline just before sleep and further decreases upon entering NREM sleep. Abnormal circadian rhythms affect body temperature and reduce sleep quality [42]. Humans regulate body temperature in response to elevated temperatures during sleep by increasing the exposed surface area [43,44]. A study of sleep in three geographically segregated human populations found that a lower ambient temperature was associated with different times of sleep onset [45]. Sleep often occurs after sunset when ambient temperature declines; warming of the hands and feet induces NREM sleep [46,47,48], and direct warming of the hypothalamus was shown to promote sleep [49]. Exposure to a warm environment was found to activate hypothalamic neurons in mice, leading to the induction of sleep [50]. According to existing studies, animals’ sleep is regulated by temperature as an environmental cue. Therefore, a foundation for using temperature to treat sleep disorders can be established by comprehending and researching the relationship between temperature and sleep.

This section may be divided by subheadings. It should provide a concise and precise description of the experimental results, their interpretation, as well as the experimental conclusions that can be drawn.

## 3. Thermoreception and Sleep Regulation in *Drosophila*

### 3.1. Thermoreception in Drosophila 

*Drosophila* and mammals have thermoreceptors that sense internal and external temperatures, to prevent physiologic injury caused by extreme cold and hot. Drosophila is an ectothermic animal, and changes in environment temperature will directly lead to changes in body temperature. *Drosophila* has sensitive thermoreceptors that can sense and activate a rapid response to temperature changes [51,52]. *Drosophila* uses distinct systems at different developmental stages to adapt to temperature variations [53,54], making it an ideal model system for the study of thermoreception.

Animals sense temperature changes through transient receptor potential (TRP) family proteins, which are cation channels present on the cell membrane and in organelles [55,56,57] that conduct Ca^2+^ influx upon activation [58,59,60]. There are 13 TPR genes in *Drosophila* [61] (Table 1), that are critical for sensing the external environment and play important roles in vision, olfaction, and thermoreception. In adult *Drosophila*, environmental temperature is detected by temperature-regulated TRP channels that are expressed in different cell types [62]. dTRPA1 is critical for detecting innocuous temperatures between 20 °C and 29 °C [63]. Anterior cell (AC) neurons [63] in the head and the hot cell neurons in the arista [64] express TRP, sense temperature, and transmit signals to different brain areas that regulate sleep behavior in *Drosophila* [34,65,66,67]. dTRPA1 is involved in locomotor behavior and synchronization of temperature cycling [68]. *Drosophila* exhibits bimodal behavior at 25 °C, with characteristic morning and evening activity peaks (M and E peaks) [69]. At physiologic temperatures (29–30 °C), the phase of M and E peaks is advanced and retarded, respectively, leading to sleep induction [70]. *Drosophila* also uses the taste receptor GR28 to sense temperature changes [71]. The painless and *pyrexia* gene encode high temperature-sensing receptors; the former is expressed in central and peripheral sensory neurons with an activation threshold of 42 °C [72], whereas the latter mediates heat-activated currents with a threshold of 40 °C [73]. *Drosophila* lacking *pyrexia* are heat intolerant, becoming paralyzed upon exposure to 40 °C for 3 min.

**Figure 1:**
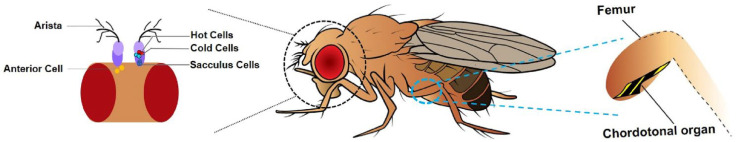
Schematic of the anatomy of thermoreception in *Drosophila*. Left: Hot cells (red) and cold cells (blue) that sense heat and cold are present peripherally in the arista. The Sacculus cells (dark blue) are located in a pouch called the Sacculus within the antennal segments (light and dark purple). Anterior cells (yellow) are found inside the brain, near the antennae. Right: The chordotonal organ (yellow-black stripes) is located at the root of the *Drosophila* leg.

Similar to other animals, adult flies have a temperature-sensing system comprising structures and cells that function in thermoreception; these include arista, which detect warm, hot, and cool temperatures [71,93] as well as anterior cell (AC) neurons in the head [63], sacculus cells in the antennae [64], and chordotonal organs in the body [94,95] (Figure 1).

dTRPA1-expressing ACs are a small group of thermally activated thermal sensors; they are considered as internal thermal sensors because they are in the brain of adult *Drosophila*, which are known to be autonomously thermosensitive [96]. Additionally, ACs integrate temperature information from peripheral sensors located in the antennae [96]. *Drosophila* achieves thermorhythmic regulation by aligning physiologic responses to ambient temperature. ACs transmit serotonin signals to small lateral ventral neurons (s-LNvs); these relay the temperature signal to dorsal neuron 2 (DN2), which regulates temperature preference rhythms. The AC-sLNv-DN2 neural circuit sets the preferred temperature before dawn and s-LNvs regulate motor activity and sleep rhythms; predawn environmental temperature may be a wake-up signal that regulates body temperature [97]. ACs have been shown detect changes in environmental temperature and release acetylcholine to activate DN1p and promote wakefulness. DN1p releases the neuropeptide CNMamide to pars intercerebralis (PI) neurons, thereby reducing the amount of sleep at night. Thus, the AC-DN1p-PI neural circuit integrates heat-sensing inputs to promote wakefulness [98].

The antenna is a temperature-sensing organ containing a variety of sensory structures that allow *Drosophila* to detect temperature changes [99]. Each arista contains brav1- and Gr28b-positive neurons that sense cold and heat [71,100]; temperature signals are transmitted by these cells to different regions of the proximal antennal protocerebrum [64,101], and then to the mushroom body [102]. In the absence of thermosensitive cells in the antenna, the normal response of DN1 neurons is attenuated, leading to disruption of sleep timing during temperature cycling [33]. Temperature signals are transmitted by pyx-expressing neurons in the antenna to ACs and then to the posterior antennal lobe [64,96], which integrates temperature-related information for transmission to higher brain centers.

Cold-sensing neurons in the antenna sense and receive signals from low-temperature stimuli. When the temperature is decreased from 25 °C to ~20 °C, these neurons relay the cold signal to the next neuron, which releases factors that inhibit the activity of sleep-promoting DN1a neurons in the brain [93].

Clock neurons in the brain are synchronized by signals from temperature-responsive peripheral tissues [103]. Loss of nocte alters the structure and function of the chordotonal organ and interferes with the synchronization of behavioral activity to temperature [94]. The *nocte* mutant flies exhibit abnormal sleep and the expression of PER and TIM in most clock neurons are abnormal during temperature cycling [33]. The nocte protein expressed in the chordotonal organs senses changes in environmental temperature and transmits signals to DNs that regulate activity and sleep by integrating light and temperature signals [104,105].

IR25a is expressed in a subpopulation of chordotonal organ neurons; loss-of-function mutants fail to synchronize with temperature cycling under both constant light and constant dark conditions. Rhythmic oscillations of TIM in DN1 and DN2 are also impaired, suggesting that these neurons receive temperature input from the chordotonal organ and are involved in circadian regulation [95].

The chordotonal organ participates in temperature sensing for circadian clock synchronization. However, direct evidence of temperature sensitivity of this organ in adult *Drosophila* is lacking, and the putative circuits connecting chordotonal organ neurons and clock neurons in the brain have yet to be described.

### 3.2. Neural Circuits That Regulate Sleep in Response to Temperature Changes

**Figure 2:**
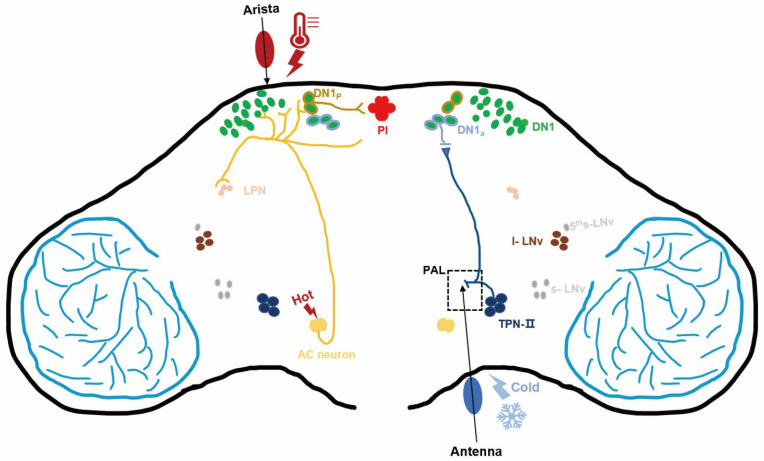
Thermoreceptor circuits in *Drosophila*. Some thermoreception and circadian neurons in the brain are shown in different colors. The antennae sense cold temperatures, and the second-order thermosensory projection neuron TPN-IIs transmit information from the posterior antennal lobe (PAL) to circadian neurons DN1a to regulate sleep in *Drosophila*. Arista and AC neurons sense warm temperatures and transmit information to some of the circadian neurons. One of the AC downstream neunon types, a subset of DN1ps (green circles with brown outline), transfers heat signals to PIs (red) to regulate sleep.

DN neurons are a heterogeneous group of neurons that can be classified into different subgroups according to gene expression and function [106,107]. DNs have a bidirectional regulatory effect on sleep, promoting both sleep [108] and wakefulness [109] (Figure 2).

Circadian clock activity in different parts of the *Drosophila* brain is studied by raising or lowering the ambient temperature. DN1p are circadian neurons that are activated and inhibited at low and high environmental temperatures, respectively. In male flies, two distinct sets of TRPA1-expressing thermosensory neurons transmit signals to DN1p at temperatures >29 °C, which influences morning sleep in response to elevated temperatures [37]. The activity of DN1a neurons is inhibited at low temperatures (18 °C). DN1a neurons release the neuropeptide CCHa1 to regulate s-LNv clock neuron activity and also receive PDF signals from s-LNv neurons; this interaction modulates sleep in *Drosophila*. Thus, DN1a-mediated temperature input may affect other clock neurons [110]. DN1p neurons can also receive high temperature signals from ACs that inhibit neuronal activity and promote wakefulness. Studies have shown that acute heating inhibits CNMa + DN1ps in an AC-independent manner, whereas prolonged heating may activate CNMa + DN1ps through heat-sensing input from AC neurons. That is, CNMa + DN1ps are activated by heat-sensing input from AC neurons to support warmth-induced arousal [98]. The evidence from these studies suggests that DN1 clock neurons serve as a “hub” for temperature input from the periphery and central sleep regulation in *Drosophila*.

The *Drosophila* neuropeptide diuretic hormone 31 (DH31) and pigment-dispersing factor receptor (PDFR) regulate nighttime temperature preference. DH31 and PDFR in DN2s regulate the preferred temperature for nighttime sleep onset [111]. DN2 neurons also receive signals from AC cells and sLNvs, with the latter signals peaking before dawn [97].

At the molecular level, temperature regulation involves thermosensitive alternative splicing of clock genes. A high frequency of excision of dmpi8, the temperature-sensitive 3′-terminal intron of the clock gene period (*per*), inhibits cold-weather sleep as more stable *per* transcript and protein is produced, leading to earlier peaks of nocturnal activity [112,113]. The dmpi8 splicing efficiency affects the transcription level of *dyw* [41]. Low temperature-dependent splicing increases *dyw* mRNA, which inhibits daytime sleep [40].

## 4. Thermoreception and Sleep Regulation in Mammals

### 4.1. Thermoreception in Mammals

TRPV1, the most widely studied temperature receptor in mammals, is expressed in the dorsal calcaneal ganglion and trigeminal ganglion (TG). High temperatures activate TRPV1 and cause a painful sensation [55,85]. *Trpv1* knockout mice have diminished or absent temperature response [86], underscoring its importance in thermoreception. TRPV2 has 50% sequence similarity to TRPV1 [114,115], is activated at 52 °C, and is primarily expressed in neurons that detect noxious thermal and mechanical stimuli [116]. Loss of TRPV2 did not impair the response of animals to heat [117], implying that TRPV2 is not required for thermoreception in adult mice. TRPV3, which is activated at 33 °C, is expressed in skin keratinocytes, sensory nerve cells, dorsal root ganglion (DRG), TG, and the brain [88,118]. TRPV4 is activated at 27 °C and is expressed in the hypothalamus, sensory neurons and skin keratinocytes [89,119]. TRPM8 was the first receptor to be identified that is activated by cold [120,121,122], and is expressed in the TG and DRG. In addition to TRP channels, ANKTM1, which senses lower temperatures, is only found in the DRG [91].

In response to heat or cold stimuli, somatosensory neurons in the skin send signals to the brain which perceives the change in temperature. In mammals, these neurons are distributed in the DRG [123,124] and are pseudounipolar with a single axon that bifurcates into two branches, one extending to the skin or viscera (detecting changes in environmental and internal core temperature, respectively) [124,125] and the other projecting to the dorsal horn of the spinal cord or spinal trigeminal nucleus in the brainstem.

DRG neurons are the primary afferent neurons of the somatosensory system and convert signals from the external and internal environment into neural activity [126,127,128]. Loss of FGF13 in sensory neurons abolishes the perception of thermal pain without affecting that of mechanical pain [129]. Likewise, ablation of sensory neurons expressing the cold receptor TRPM8 resulted in an inability to perceive cold [130,131,132]. Thus, different temperature receptors in sensory neurons sense temperature changes.

There are four main types of sensory neuron that can be categorized as Aα, Aβ, Aδ, and C fibers, or classified according to the type of stimulus that is detected (mechanical or thermal). Aδ and C fibers include the sensory nerves involved in thermosensation [124,133]. Branches of Aδ and C fibers are widely distributed in the skin and sense different ranges of temperature [134,135,136,137]; this information is encoded as an action potential and transmitted to another axon branch. Aδ axon terminals are distributed in layers I and V of the dorsal horn, while those of C fibers are distributed and form synapses in layers I and II. Temperature information is transmitted via the ascending spinothalamic tract to the thalamus and somatosensory cortex, where temperature perception occurs [125]. Temperature information is also transmitted to the pre-optic area (POA) of the hypothalamus via lateral parabrachial neurons [138,139].

Neurons in the DRG sense temperature changes in most areas of the body, while primary sensory neurons of the TG are mainly distributed in the head and face [140,141,142,143]. The TG is a sensory ganglion that expresses various neuropeptides and signaling molecules and senses temperature changes with a higher temperature threshold and slower action potential conduction velocity than the DRG [142,144].

Warm, hot, and cold stimuli are sensed by different groups of neurons in the TG [121,145,146]. Hypothermia induces the activation of about 15% of non-cold-sensing neurons in the TG [147] that are also involved in temperature perception. The TG contains distinct populations of spinothalamic projection neurons that transmit temperature information to the primary somatosensory cortex [138].

### 4.2. Association between Temperature and Sleep Regulation

Mammals, unlike *Drosophila*, are homeotherms, which means that their core body temperature is kept within a specific range. The brain regulates body temperature by boosting heat generation or heat dissipation based on temperature information transmitted from the skin and organs by skin receptors. Mammals’ core and skin temperatures are frequently separated into two categories. Animals’ skin, which is on the outside of them, frequently varies in response to variations in the surrounding temperature, whereas the internal organs and central nervous system have temperatures that are more or less constant [125,148]. Internal organs, the brain, and the spinal cord all keep track of variations in core temperature, and skin receptors send information about environmental temperature to the brain, which regulates body temperature by boosting either heat generation or heat dissipation. The inner core and the skin both communicate temperature data to the POA. Clinical studies have demonstrated that excessively high or low environmental temperature can affect the amount and quality of sleep [149,150,151,152,153].

The hypothalamus receives signals pertaining to temperature changes and participates in thermoregulation; damage to the hypothalamus can also seriously affect sleep. Neurons involved in sleep and temperature regulation are located in the POA [154,155,156] and their activity increases with temperature [157]. The POA is divided into lateral (lPOA), median (MnPOA), and medial (mPOA) nuclei. Activation of GABAergic or galaninergic neurons in the POA promotes sleep [158,159], whereas activation of glutamatergic neurons promotes wakefulness [158,160]. The mPOA is involved in sleep and temperature regulation [161,162]; impaired mPOA function in rats decreased sleep, increased brain temperature, and abolished the ability of the rats to regulate body temperature in a cold environment [161,163]. Elevated orexin levels in the mPOA of lactating rats promoted wakefulness and increased body temperature [164]. Upon exposure to a warm temperature, thermoreceptors in the skin transmit signals to glutamatergic neurons in the LPB nucleus, which activates glutamatergic neurons in the MnPOA and mPOA. MnPOA GABAergic neurons inhibit wakefulness-inducing neurons to promote sleep, while mPOA GABAergic neurons promote hypothermia [50,165]. The lPOA and mPOA regulate sleep in different ways; disruption of the former in rats was shown to reduce sleep while brain temperature was unaffected, although the rats exhibited defects in thermal defense behaviors [161].

During sleep, the temperature of the skin and brain changes. During NREM sleep, the brain and skin temperature decrease; during REM, brain temperature increases. It is suggested that body temperature and sleep stage are closely related. Recording changes in brain temperature while recording neuronal activity found that brain temperature was closely related to changes in sleep at different scales [166,167].

## 5. Outlook and Discussion

For *Drosophila* and mammals, temperature provides basic information regarding the environment. A major advance in our understanding of temperature perception was the identification of distinct thermoreceptors in different species and the cloning and characterization of molecular thermosensors. *Drosophila* is highly sensitive to temperature changes, and it also has a limited number of neurons and multiple genetic tools available, making it amenable to the study of the relationship between temperature and sleep. The current study shows that the transmission of temperature signals in *Drosophila* has been elucidated. There might, however, yet be undiscovered ways for sensing temperature. The temperature loop can be more clearly described by studying the transmission of temperature signals in different ways [168,169]. It also lays the foundation for exploring the neural circuits of temperature regulation and sleep.

Sleep and body temperature are also closely related in mammals, with both environmental and body temperatures affecting sleep. The POA receives temperature information from the skin and participates in temperature and sleep regulation. Multiple distinct neuron types with different properties and molecular profiles that regulate sleep or body temperature have been identified in the POA. The extent of overlap between temperature-sensing and sleep-regulating POA neuron populations and associated neural circuits are unknown. The current study demonstrates the heterogeneity of POA’s neurons and suggests that various neurons may control various biological processes. Recent single-cell sequencing techniques classify POA neurons more precisely. From there, researchers can manipulate a particular type of POA neuron using light activation or virus injection to better understand how different POA neuron types work in concert to control body temperature and sleep. Most of the current research on sleep in mammals is conducted using mice; as they are nocturnal animals, it is unclear whether the mechanisms of temperature and sleep regulation reflect those in humans. Examining how temperature affects sleep in other species can provide insight into whether the mechanisms that have been described in model organisms are conserved across all animals.

In recent years, researchers have discovered and characterized the networks and neural circuits involved in temperature transmission. However, our understanding of animal thermal sensations still lags behind our understanding of other senses such as sight, smell, and so on. In *Drosophila* and mammals, many key temperature-sensing neurons and thermoreceptor molecules have not been discovered, and little is known about the roles of known molecules in sleep regulation. Mammalian research advances slowly due to the enormous number of neurons and limited technological capabilities. Given the complexity of mammals’ thermoregulatory systems and temperature sensing networks, it is difficult to fully understand their regulatory processes in the present studies. This makes it challenging to determine how temperature regulation affects sleep. It is anticipated that as technology advances, new neurological pathways controlling sleep through temperature will be discovered, allowing humans to alter the environment’s temperature to enhance sleep quality.

## Figures and Tables

**Table 1:** Transient receptor potential channels in *Drosophila* and mammals.

Channels	Function	Reference
*Drosop* *h* *ila*		
dTRPA1	Monitoring temperature fluctuations	[74]
	A isoform: >26 °C	[75,76,77]
	B isoform: >34 °C	[78,79,80,81]
TRPC	Cold avoidance (10 °C)	[74]
TRPV	Thermotactic response to cold temperature	[82]
TRPP	Thermotactic response to cold temperature	[64]
Painless	Avoidance of noxious heat (>40 °C)	[72,83]
Pyrexia	Noxious heat resistance (40 °C)	[73,84]
Mammals		
TRPV1	Avoidance of noxious heat (>43 °C)	[85,86]
TRPV2	Avoidance of noxious heat (>52 °C)	[87]
TRPV3	Avoidance of noxious heat (>33 °C)	[88]
TRPV4	Avoidance of noxious heat (27–42 °C)	[89]
TRPM8	Distinguishing between low and medium temperatures (<23–28 °C)	[90]
ANKTM1	Cool temperature avoidance (<18 °C)	[91,92]

## Data Availability

Not applicable.
